# Living Alone in Late Life

**DOI:** 10.1093/geroni/igab046.1844

**Published:** 2021-12-17

**Authors:** Jacqueline Torres

**Affiliations:** University California San Francisco, San Francisco, California, United States

## Abstract

Approximately one third of older adults in the United States (US) and 13% of older adults in Mexico live alone. In both countries, the prevalence of living alone is higher for women and increases with advanced age; in the US, an estimated 4.3 million older adults continue to live alone with cognitive impairment or dementia. We will present research from the US and Mexico on the receipt of long-term services and supports and unmet needs for care among older adults living alone, including with cognitive impairment, as well as factors that may modify these outcomes. For the US, we will describe recent findings about the health, health care, and caregiving outcomes of older adults living alone vs. living with others during the COVID-19 pandemic.

